# A comprehensive introduction to the genetic basis of non-syndromic hearing loss in the Saudi Arabian population

**DOI:** 10.1186/1471-2350-12-91

**Published:** 2011-07-04

**Authors:** Faiqa Imtiaz, Khalid Taibah, Khushnooda Ramzan, Ghada Bin-Khamis, Shelley Kennedy, Bashayer Al-Mubarak, Daniah Trabzuni, Rabab Allam, Abeer Al-Mostafa, Sameera Sogaty, Abdulmoneem H Al-Shaikh, Saeed S Bamukhayyar, Brian F Meyer, Mohammed Al-Owain

**Affiliations:** 1 Department of Genetics, King Faisal Specialist Hospital & Research Centre, PO Box 3354, Riyadh 11211, Saudi Arabia; 2 ENT Medical Centre, PO 340507, Riyadh 11333, Saudi Arabia; 3 Department of Otololaryngology, King Faisal Specialist Hospital & Research Centre, PO Box 3354, Riyadh 11211, Saudi Arabia; 4 Ontario Newborn Screening Program, Children's Hospital of Eastern Ontario 401 Smyth Road, Room 3127A Ottawa, Ontario K1H 8L1, Canada; 5 Medical Genetics Unit, King Fahad Hospital, PO Box 8488, Jeddah 21196, Saudi Arabia; 6 ENT Department, King Fahad Hospital, PO Box 8488, Jeddah 21196, Saudi Arabia; 7 Audio-Vestibular Medicine Unit, King Fahd Hospital, PO Box 8488, Jeddah 21196, Saudi Arabia; 8 Department of Medical Genetics, King Faisal Specialist Hospital & Research Centre, PO Box 3354, Riyadh 11211, Saudi Arabia; 9 College of Medicine, Al-Faisal University, PO Box 50927, Riyadh 11533, Saudi Arabia

## Abstract

**Background:**

Hearing loss is a clinically and genetically heterogeneous disorder. Mutations in the *DFNB1 *locus have been reported to be the most common cause of autosomal recessive non-syndromic hearing loss worldwide. Apart from *DFNB1*, many other loci and their underlying genes have also been identified and the basis of our study was to provide a comprehensive introduction to the delineation of the molecular basis of non-syndromic hearing loss in the Saudi Arabian population. This was performed by screening *DFNB1 *and to initiate prioritized linkage analysis or homozygosity mapping for a pilot number of families in which *DFNB1 *has been excluded.

**Methods:**

Individuals from 130 families of Saudi Arabian tribal origin diagnosed with an autosomal recessive non-syndromic sensorineural hearing loss were screened for mutations at the *DFNB1 *locus by direct sequencing. If negative, genome wide linkage analysis or homozygosity mapping were performed using Affymetrix GeneChip^® ^Human Mapping 250K/6.0 Arrays to identify regions containing any known-deafness causing genes that were subsequently sequenced.

**Results:**

Our results strongly indicate that *DFNB1 *only accounts for 3% of non-syndromic hearing loss in the Saudi Arabian population of ethnic ancestry. Prioritized linkage analysis or homozygosity mapping in five separate families established that their hearing loss was caused by five different known-deafness causing genes thus confirming the genetic heterogeneity of this disorder in the kingdom.

**Conclusion:**

The overall results of this study are highly suggestive that underlying molecular basis of autosomal recessive non-syndromic deafness in Saudi Arabia is very genetically heterogeneous. In addition, we report that the preliminary results indicate that there does not seem to be any common or more prevalent loci, genes or mutations in patients with autosomal recessive non-syndromic hearing loss in patients of Saudi Arabian tribal origin.

## Background

Deafness, the inability to hear, is the most common sensory deficit in human populations with both genetic and environmental etiologies. It is estimated that it affects 1 in 1000 child births of which approximately 60% cases are attributed to genetic factors [1]. Hearing impairment is clinically and genetically heterogeneous. Impaired auditory function can be the only clinical manifestation (non-syndromic forms of deafness) or can be associated with other symptoms or anomalies (syndromic forms of deafness). It has been estimated that at least 300 human protein-coding genes are involved in the hearing process [2]. During the last decade, many deafness loci and the underlying genes have been identified at a rapid rate.

The main pattern of inheritance in severe childhood deafness is autosomal recessive (over 75%) while autosomal dominant (12-24%), X-linked (1-3%) and mitochondrial is also involved [3]. In general, recessive deafness is more likely to be more severe than dominant deafness because it is generally profound, prelingual and fully penetrant whereas dominant deafness is often progressive, post lingual and is frequently observed clinically as unilateral or mild bilateral deafness [4]. In addition, recessively inherited diseases are more prevalent in populations where consanguineous marriages are common, like in the kingdom of Saudi Arabia. The same effect is observed for recessively inherited cases of deafness.

Autosomal recessive non-syndromic hearing loss (ARNSHL) is the most frequent cause of hereditary deafness and often exhibits the most severe hearing phenotype. Presently, 85 recessive deafness (*DFNB*) loci have been registered and 35 of the corresponding genes have been identified as documented on the Hereditary Hearing Loss Homepage http://hereditaryhearingloss.org. Interestingly, even though a substantial number of genes are known to cause ARNSHL, mutations at the first identified *DFNB1 *locus, account for up to 50% of all cases with this diagnosis in various populations. *DFNB1 *contains the *GJB2 *and *GJB6 *genes that respectively code for connexin 26 (C×26) and connexin 30 (C×30), which are gap junction binding proteins, most abundantly expressed in the cochlea. Although over 100 mutations have been reported in *GJB2 *(The Connexin-deafness homepage: http://davinci.crg.es/deafness), a single mutation, c.35delG, is the most common cause of ARNSHL and can account for up to 85% of *DFNB1 *in various populations [5]. *DFNB1 *also harbors the second most common ARNSHL causing mutation, a 309 kb deletion of *GJB6*, often found *in trans *with mutations in *GJB2*, giving rise to a digenic inheritance of this form of hereditary hearing loss.

In view of the extensive studies conducted worldwide, the major objective of this study was to begin to comprehensively delineate the genetic basis of ARNSHL in individuals of Saudi Arabian origin. To achieve this objective, individuals segregating with severe to profound, non-syndromic congenital deafness with an autosomal recessive mode of inheritance were identified from different cities of Saudi Arabia and initially screened for mutations encompassing the *DFNB1 *locus.

The second aim was to initiate prioritized linkage analysis (in families of three or more affected individuals) and homozygosity mapping (in families with two affected sib-pair individuals) of other known deafness loci for families in which *DFNB1 *has been excluded. This further bi-directional approach was necessary due to the vast heterogeneity and complexity of the number of genes known and unknown causing hereditary deafness. In this study, using SNP-based genotyping arrays, candidate genes were selected and screened in the chromosomal region that generated the highest LOD score (multipoint linkage analysis) or in the largest region of homozygosity shared by the affected sib-pairs that was not seen in the parents or unaffected siblings if applicable. This genome-wide homozygosity mapping analysis approach was used in this study in particular, as it assumes that individuals affected with an autosomal recessive disease, born from parents of a consanguineous marriage are very likely to be homozygous for the pathogenic mutation and for a substantial number of SNPs surrounding it [6].

The overall results of this study are highly suggestive that underlying molecular basis of autosomal recessive non-syndromic deafness in Saudi Arabia is very genetically heterogeneous. In addition, we report that from the results of our pilot study there does not seem to be any common or more prevalent loci, genes or mutations in patients with ARNSHL originating from the kingdom.

## Methods

### Subjects

All of the individuals (patients and family members) who participated in this study provided an approved informed consent form, which adhered to institutional (King Faisal Specialist Hospital; RAC# 2040039) and international guidelines as outlined by the Declaration of Helsinki.

### Clinical evaluation

Detailed medical histories were obtained for all of the enrolled affected individuals regarding time and age of onset of hearing loss (pre-lingual, post-lingual), severity of hearing loss (mild, moderate or profound) and in order to exclude syndromic abnormalities, environmental causes for hearing loss and an autosomal dominant inheritance pattern of hearing loss. Pure tone audiometry tests for air and bone conduction were performed on all affected individuals. Evaluation of vestibular and ocular function was performed to exclude suspected forms of syndromic deafness.

### Sample Collection and DNA Extraction

Whole blood samples (10 ml) for molecular genetic analysis were obtained from affected patients, their parents and unaffected siblings (if available). Genomic extraction of DNA was performed using the standard salting-out method [7].

### Mutation Detection in *GJB2 *and *GJB6*

Mutation screening by direct dideoxy chain-termination sequencing of the entire coding region of PCR generated amplicons (using standard PCR conditions) was performed for both the *GJB2 *and *GJB6 *genes in all affected individuals in the study using an ABI Prism Big Dye Terminator v3.1 Cycle Sequencing Kit following the manufacturer's instructions and processed on a MegaBACE 1000 DNA Analysis System (Molecular Dynamics; Sunnyvale, CA, USA). Sequence analysis was performed using the SeqMan 6.1 module of the Lasergene (DNA Star Inc. WI, USA) software package, then compared to the reference GenBank sequence (accession number: *GJB2*; NM_004004 and GJB6; NM_001110219). Numbering commenced with the A of the ATG initiation codon as +1.

### Linkage analysis

SNP-based genotyping was performed using the Affymetrix GeneChip^® ^Human Mapping 250 K and 6.0 Arrays (Affymetrix, Santa Clara, CA, USA) as per the user's manual. The genotypes of SNPs were called using Affymetrix GCOS 1.4 software, which generated an overall average SNP call rate of 97% and were further sorted per chromosome and by physical position. The Allegro module of the Easy Linkage software package was used to calculate multipoint logarithm of odds (LOD) scores, with the parameters that assume a disease model with an autosomal-recessive mode of inheritance with 100% penetrance and a disease allele frequency of 0.0001.

The resulting multipoint linkage analysis of the affected patients, their unaffected siblings, and their parents which resulted in the highest maximum LOD score was investigated. Any known deafness causing gene(s) located in this interval were assumed the likely disease-causing candidate and the patients were screened by direct sequencing of the coding sequence for the particular gene(s).

### Homozygosity mapping

The resulting SNP-based genotyping data (as in Linkage Analysis) was analyzed to detect regions of homozygosity using CNAG 3.0 and GTConsole Version 3.0.1 (Affymetrix, Santa Clara, CA, USA) software packages. Conventionally, regions of homozygosity are defined as fragments where SNPs are homozygous for a stretch of consecutive alleles in affected individuals and heterozygous or homozygous for the other allele in unaffected members of the same family. As previously reported by researchers using this approach [8], it is advantageous to identify and initially screen candidate genes present in the largest region of homozygosity shared between affected family members, however this was not true in all families that were analyzed in this manner.

### Mutation Detection in *MYO15A, TMPRSS3, TRIC, LHFPL5, TMC1*

After linkage and homozygosity mapping analysis, genomic DNA from relevant individuals was amplified using standard PCR conditions with intronic primers that were designed to flank (50-100 bp) each of coding exons of *MYO15A, TMPRSS3, TRIC, LHFPL5 *and *TMC1 *as mentioned previously (primer sequences and conditions are available on request).

### RT-PCR

To determine the effect of the novel splice-acceptor site mutation (IVS12-1 g>c) in family KT1 with respect to mRNA splicing, a fragment comprising exon 11 and 12 of *TMPRSS3 *was amplified using RT-PCR with mRNA isolated from peripheral blood lymphocytes. Preparation of the cDNA was carried out using the iScript™ cDNA synthesis kit and random primers (Applied Biosystems, Carlsbad, CA). One microgram of total RNA from the patient and a normal control was reverse transcribed using an aliquot of a reverse transcription reaction containing 1 × PCR buffer, 2.5 mM MgCl_2_, 0.2 mM of each dNTP, and 0.5 U AMV reverse transcriptase (Roche Diagnostics GmbH, Mannheim, Germany). 10 μl of cDNA was amplified by PCR using the 5' primer (TCC AAC AAG ATC TGC AAC CA) and the 3' primer (ATC CAG TCC AGG AAG GAG GT) using standard conditions. PCR products were then evaluated on 2% agarose gel and were purified using the QIAquick PCR Purification Kit according to the manufacturer's instructions (Qiagen, Germantown, MD) and then sequenced as described above.

## Results

*GJB2 *and *GJB6 *were screened from affected individuals with pre-lingual severe to profound ARNSHL from a total of 130 families, which included both multiple affected and sporadic cases. *DFNB1 *mutations were identified in only 4 of the 130 families. Patients from two of the families were homoallelic for the common c.35delG mutation in *GJB2*. One patient was homozygous for the R32H mutation and the fourth patient was homozygous for W77R, both of which have been previously reported in *GJB2 *(Table 1). No other mutations have been found in *GJB2 *or *GJB6 *(including the 309-kb deletion) in this largest cohort of patients from this population. Therefore, our results show that mutations in *DFNB1 *account for only 3% of ARNSHL in patients of Saudi Arabian tribal ethnicity.

**Table 1:** Summary of mutations in various known deafness genes identified in ARNSHL patients from Saudi Arabia.

FAMILY/SIBSHIPS	MODE OF ANALYSIS	REGION OF INTEREST	GENE OF INTEREST	NUCLEOTIDE CHANGE	PROTEIN CHANGE	REFERENCE
**NSHD28/69**	*DFNB1 *SCREENING	NA	*GJB2*	c.35delG	Frameshift	[10]

**NSHD74**	*DFNB1 *SCREENING	NA	*GJB2*	95 G>A	p.R32H	[11]

**KT7**	*DFNB1 *SCREENING	NA	*GJB2*	c.229T>C	p.W77R	[12]

**SS16**	Linkage (LOD SCORE)	Chr 17p12-q11.2	*MYO15A*	c.1047C>A	p.Y349X	This study

**KT1**	Linkage (LOD SCORE)	Chr 21q22.2-q22.3	*TMPRSS3*	IVS12-1 g>c	Splicing error	This study

**TA12**	Homozygosity (GT console)	Chr 5q11.2-q13.3	*TRIC*	c.1498C>T	p.R500X	[13]

**SS3**	Homozygosity (GT console)	Chr 6p24.3-p12.3	*LHFPL5*	c.1A>G	p.M1V	[8]

**SS17**	Homozygosity (CNAG)	Chr 9p13.3-q21.13	*TMC1*	c.100C>T	p.R34X	[14]

### Linkage analysis and homozygosity mapping in 5 Families with ARNSHL

**Figure 1:**
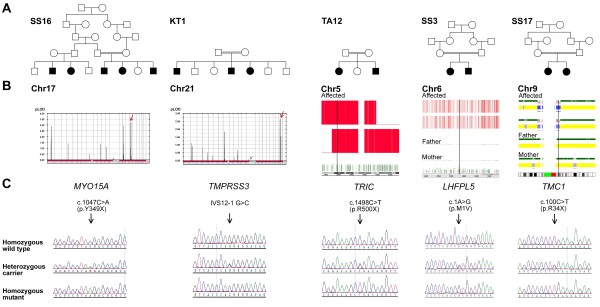
**Family pedigrees, linkage analysis/homozygosity mapping, and mutation data**. A) Pedigrees of five Saudi families segregating with autosomal recessive prelingual profound hearing loss. B) Linkage analysis showing LOD scores of 3.3 on chromosome 17 and 2.7 on chromosome 21 for families SS16 and KT1, indicating linkage to *MYO15A *and *TMPRSS3* genes, respectively. Homozygosity mapping with GT console is shown for *TRIC *and *LHFPL5 *as genes of interest for families TA12 and SS3, respectively. For family SS17, homozygosity mapping with CNAG shows the region of homozygosity on chromosome 9 where *TMC1 *resides. C) Sequencing chromatogram of wild type, heterozygous and mutant alleles of respective deafness genes for a normal control, a carrier and affected member of each of the families.

The exclusion of *DFNB1 *as a major cause of ARNSHL allowed us to accelerate our efforts with respect to linkage analysis and homozygosity mapping. 70 of the 130 families that were enrolled in the study had 2 or more affected individuals, the remaining being made up of sporadic cases. Initially as a pilot study, patients from five families were selected for analysis on the basis that the parents confirmed consanguineous marriages and that the non-syndromic hearing loss was consistent with a recessive mode of inheritance. Two families of 3 affected individuals were subjected to LOD score calculation by linkage analysis and the remaining 3 families with two affected individuals were analyzed by homozygosity mapping (Figure 1). Multipoint linkage analysis of the first family generated a LOD score of 2.5 on chromosome 17p12-p11.2 in which *MYO15A *was selected as a candidate within a 6.0 Mb linkage interval. Similar analysis on the second family resulted in a maximum LOD score of 2.7 (Figure 1) and identified a disease locus on chromosome 21q22.3, which spanned approximately 1.5 Mb. *TMPRSS3 *was selected as a potential candidate in this region. Homozygosity mapping analysis in the remaining 3 sibships generated multiple regions of homozygosity that was shared only among affected individuals. But as previously discussed, any known *DFNB *genes that were located in the longest stretch of homozygosity were chosen for sequencing in the respective family. On this basis, *TRIC, LHFPL5*, and *TMC1 *were selected to be directly sequenced from the genomic DNA of all members in the particular family that affected patients were homozygous for the interval containing the applicable gene.

### Mutation Screening in *MYO15A, TMPRSS3, TRIC, LHFPL5, TMC1*

Direct sequencing in both the forward and reverse directions of each candidate gene that the respective affected siblings linked to, revealed the presence of homozygous mutations in all of the five genes (Figure 1). The mutations segregated with the disease phenotype where applicable, i.e. parents were heterozygous carriers for the mutation and unaffected siblings were either heterozygous carriers or wild-type normal. Two of the five mutations found were novel (Table 1). Neither of the novel mutations were found in 300 ethnically matched normal controls, which indicated that these variants were not population-based polymorphisms.

**Figure 2:**
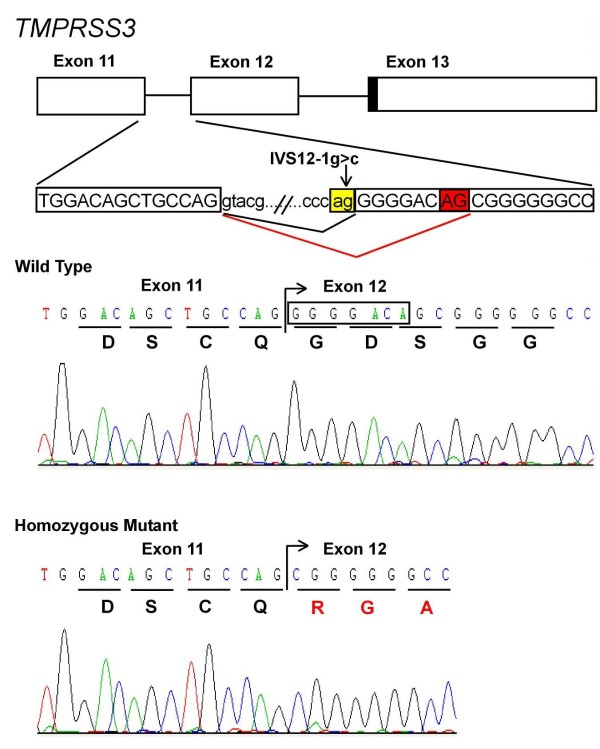
**Schematic presentation for the genomic localization and the sequence of splice site mutation**. The IVS12-1 g>c mutation (indicated by an arrow) creates a 5' cryptic acceptor site (AG boxed in red) 8 bp downstream in exon 12 instead of a normal acceptor site (ag boxed in yellow). Sequencing of the mutant transcript shows that cryptic splice site leads to a frameshift and incorporation of faulty amino acids as compared to the wild type.

RT-PCR products were utilized to establish the effect of the novel splice-acceptor site mutation (IVS12-1 g>c) in the family KT1 with respect to mRNA splicing in *TMPRSS3*. Sequencing results provided the evidence that the patients which are homozygous for a novel intronic splice-acceptor site mutation shows the activation of cryptic splice site 8 bp downstream in the exon 12 leading to a frameshift and incorporation of faulty amino acids and hence confirms that this mutation does indeed affect normal splicing and had a major deleterious impact on the structure of the *TMPRSS3 *mRNA (Figure 2).

## Discussion

Our results strongly indicate that *DFNB1 *only accounts for 3% of ARNSHL in the Saudi population (Table 1). This percentage is significantly lower than a recent study by Al-Qahtani et al [9] who reported that the total mutations in *GJB2 *account for 15.59% in their cohort of patients from the western province of Saudi Arabia. However, this number is most likely elevated compared to our results as they reported *GJB2 *mutation findings on patients of different ethnic origins, which they also suggest may be a consequence of migration and settlement in the geographical area of where their study was conducted. All of the patients in this study were of Saudi Arabian tribal origin and therefore, we believe that 3% is a more accurate estimation of the contribution of *DFNB1 *in ARNSHL in individuals ethnically originating from the Kingdom.

Three of the five mutations identified by direct sequencing of known deafness genes in the candidate regions identified by linkage analysis/homozygosity mapping in 5 families in which *DFNB1 *had been excluded have been reported previously (Table 1). The novel nonsense mutation detected in *MYO15A *in family SS16 results in the premature truncation at amino acid position 349 located in the first domain (N-terminal extension) of the myosin XVa protein, which is normally comprised of 3530 amino acids. The second novel mutation, a splice-acceptor site disruption (IVS12-1 g>c) in the *TMPRSS3 *gene in family KT1 was confirmed by RT-PCR to cause abnormal splicing by activation of a cryptic splice site resulting in a frameshift and incorporation of faulty amino acids in this serine protease.

Consanguineous families are a powerful resource for genetic linkage studies of recessively inherited hearing impairment. So far, family based analyses have proven that this methodology is successful and the results have shown that the hearing loss in five separate families was caused by five different genes (Table 1), thus tentatively confirming the already well-established finding of genetic heterogeneity in ARNSHL worldwide is in fact analogous in the Saudi population. From the combined approach and data of the current study, it is not possible to exclude the possibility of more prevalent ARNSHL-causing genes in this population that have not yet been identified. However, it is pertinent to highlight that this is a pilot study and the findings discussed here will be validated by our future direction once results are obtained from further similar family-based analyses, in addition to whole exome/genome sequencing of our large number of families with a single affected proband that are not amenable to analysis using the same linkage and homozygosity mapping methodology detailed in this preliminary report.

## Conclusion

In conclusion, *DFNB1 *is a very minor cause of ARNSHL in individuals of Saudi Arabian tribal origin. By using the effective bi-directional approach of linkage analysis and homozygosity mapping that we have initiated, we will be able to identify the most common forms of hereditary hearing loss, their incidence and distribution in the Saudi population. The benefit of this study will hopefully provide the foundation for knowledge and awareness through screening of carrier status and genetic counselling, thereby having a major impact upon early intervention for and prevention of hereditary hearing loss.

## Competing interests

The authors declare that they have no competing interests.

## Authors' contributions

FI was primarily responsible for the design, molecular genetic studies, data interpretation, drafting and finalizing the manuscript; KT and MO were primarily responsible for clinical support, clinical evaluation and sample collection; MO also participated in editing of the manuscript; KR participated in molecular genetic studies and result databasing and formatting, GBK was responsible for audiological evaluation; BAM, DT, RA and AM assisted in carrying out molecular genetic studies; participated in methodology; SS, SSB and AHS contributed with sample collection, ENT and genetic evaluation; BFM participated in design, data interpretation and final editing of the manuscript. All authors have read and approved the final manuscript.

## Pre-publication history

The pre-publication history for this paper can be accessed here:

http://www.biomedcentral.com/1471-2350/12/91/prepub
